# Clinical and Laboratory Factors Associated with Serious Infections in Multiple Myeloma: A Real-World Study

**DOI:** 10.3390/curroncol33100615

**Published:** 2026-10-14

**Authors:** Aslı Odabaşı Giden, Onur Şahin, Duzgun Ozatli

**Affiliations:** 1 Department of Hematology, Ordu State Hospital, Ordu 52200, Turkey; 2 Department of Hematology, Ondokuz Mayıs University, Samsun 55139, Turkey

**Keywords:** multiple myeloma, serious infection, dialysis, treatment response, neutrophil-to-lymphocyte ratio, intravenous immunoglobulin

## Abstract

**Background:** Serious infections remain a major cause of morbidity and treatment disruption in patients with multiple myeloma (MM). Identifying readily available clinical factors associated with infectious complications may facilitate risk-adapted supportive care. This study evaluated clinical and laboratory factors associated with serious infection in patients with MM, including the potential association of baseline neutrophil-to-lymphocyte ratio (NLR) with subsequent serious infectious complications. **Methods:** This retrospective multicenter cohort study included 104 patients with MM. Baseline demographic, disease-related, treatment-related, and laboratory characteristics were evaluated. Serious infection was defined as an infectious episode requiring hospitalization. Infectious events were recorded throughout follow-up, including those occurring during second-line and subsequent therapies. Associations with serious infection were assessed using univariate and multivariable binary logistic regression analyses. The primary multivariable model included age, International Staging System (ISS) stage, dialysis requirement at diagnosis, treatment response, and baseline NLR. **Results:** Serious infections occurred in 39 patients (37.5%). Dialysis requirement at diagnosis (30.8% vs. 4.6%, *p* = 0.001), failure to achieve treatment response (51.3% vs. 7.7%, *p* < 0.001), hypogammaglobulinemia (48.7% vs. 29.2%, *p* = 0.046), and IVIG administration (76.9% vs. 46.2%, *p* = 0.002) were more frequent among patients with serious infection. Baseline NLR did not differ significantly between patients with and without serious infection. In the multivariable model, dialysis requirement at diagnosis (adjusted OR 8.26, 95% CI 1.81–37.68; *p* = 0.006) and failure to achieve treatment response (adjusted OR 16.77, 95% CI 4.37–64.31; *p* < 0.001) were independently associated with serious infection, whereas age, ISS stage, and baseline NLR were not. **Conclusions:** Serious infections were common in this real-world MM cohort. Dialysis requirement at diagnosis and failure to achieve treatment response were independently associated with serious infection, whereas baseline NLR was not independently associated with serious infectious complications. These findings suggest that clinical characteristics reflecting renal dysfunction and treatment response may be useful for identifying patients at increased risk of serious infection. The observed association between IVIG administration and serious infection should be interpreted cautiously because of potential confounding by indication and reverse causality.

## Simple Summary

Serious infections are an important complication in patients with multiple myeloma and may lead to hospitalization, interruption of cancer treatment, and poor clinical outcomes. Identifying patients at increased risk may help clinicians provide closer monitoring and preventive supportive care. In this multicenter real-world study of 104 patients with multiple myeloma, we investigated clinical and laboratory factors associated with serious infections requiring hospitalization. Serious infections occurred in 37.5% of patients. The need for dialysis at the time of diagnosis and failure to achieve a response to myeloma treatment were independently associated with serious infection. In contrast, the baseline neutrophil-to-lymphocyte ratio, a readily available marker of systemic inflammation, was not independently associated with serious infection. These findings suggest that clinical characteristics reflecting renal impairment and treatment response may be useful for identifying patients who require closer infection surveillance and individualized supportive-care strategies.

## 1. Introduction

Multiple myeloma (MM) is a clonal plasma cell malignancy characterized by the uncontrolled proliferation of terminally differentiated plasma cells within the bone marrow, resulting in progressive bone marrow failure, osteolytic bone disease, renal dysfunction, anemia, hypercalcemia, and profound immune dysregulation. MM accounts for approximately 10% of all hematologic malignancies and remains the second most common hematologic cancer worldwide. Over the past two decades, the therapeutic landscape of MM has evolved remarkably with the introduction of proteasome inhibitors, immunomodulatory drugs, anti-CD38 monoclonal antibodies, antibody-drug conjugates, bispecific antibodies, and chimeric antigen receptor (CAR) T-cell therapies, leading to unprecedented improvements in response rates, progression-free survival, and overall survival. Despite these advances, MM remains an incurable disease characterized by repeated relapses, progressive immune dysfunction, and cumulative treatment-related toxicity. Consequently, disease-related complications continue to represent major determinants of morbidity, mortality, healthcare utilization, and impaired health-related quality of life, highlighting the continuing need for optimized supportive care alongside effective anti-myeloma treatment [1,2,3].

Patients with MM exhibit increased susceptibility to bacterial, viral, fungal, and opportunistic infections, particularly during the first year after diagnosis and following the initiation of anti-myeloma therapy. This increased vulnerability results from the combined effects of disease-related immune dysfunction, impaired humoral and cellular immunity, immunoparesis, bone marrow failure, and cumulative immunosuppression induced by anti-myeloma treatment. Population-based studies have consistently demonstrated that infections account for a considerable proportion of hospitalizations, treatment interruptions, and infection-related mortality among patients with MM [4,5].

The spectrum of infectious complications has further evolved with the widespread use of contemporary anti-myeloma therapies. Although novel agents have substantially improved disease control and survival, treatment-related immune dysfunction, prolonged lymphopenia, hypogammaglobulinemia, and delayed immune reconstitution may further increase susceptibility to severe and recurrent infections. Consequently, prevention, early recognition, and individualized management of infectious complications have become essential components of modern MM care, emphasizing the need for accurate risk-stratification strategies capable of identifying high-risk patients before clinically significant infectious events occur [5,6].

Hypogammaglobulinemia and immunoparesis are hallmark immunological abnormalities in MM and represent major determinants of recurrent and severe infections. Suppression of uninvolved immunoglobulins may occur at diagnosis and typically becomes more pronounced with disease progression and repeated exposure to anti-myeloma therapies, resulting in impaired humoral immune responses and diminished protection against encapsulated bacterial pathogens. Consequently, recurrent respiratory tract infections, severe bacterial infections, and infection-related hospitalizations remain common even among patients who achieve satisfactory hematologic responses.

Intravenous immunoglobulin (IVIG) replacement therapy has been shown to reduce the incidence of serious bacterial infections in selected patients with recurrent infections and profound hypogammaglobulinemia and is increasingly recognized as an important component of supportive care in MM. Nevertheless, current recommendations advocate individualized rather than routine IVIG administration, taking into account infection history, serum immunoglobulin concentrations, and the overall clinical and immunological status of each patient. Despite these recommendations, universally accepted criteria for identifying patients who are most likely to benefit from IVIG therapy are still lacking, highlighting an important unmet need for objective and readily applicable clinical predictors to guide supportive treatment decisions [7,8,9].

Despite increasing awareness of infectious complications in MM, accurately identifying patients at the greatest risk remains challenging. Several demographic, disease-related, and laboratory variables, including advanced age, advanced disease stage, renal dysfunction, hypoalbuminemia, lymphopenia, and hypogammaglobulinemia, have been associated with an increased susceptibility to infections. However, these individual factors have demonstrated limited predictive performance when used in isolation, and no universally accepted risk stratification model is currently available for routine clinical practice. Consequently, there is growing interest in identifying simple, inexpensive, and readily accessible biomarkers that can improve early risk assessment, facilitate individualized supportive care, and assist clinicians in selecting patients who may benefit from intensified infection surveillance and preventive interventions [4,5,8,9].

Among the candidate biomarkers proposed for risk stratification, the neutrophil-to-lymphocyte ratio (NLR) has emerged as a simple, inexpensive, and readily available indicator of the balance between systemic inflammation and host immune competence. Derived from routine complete blood counts, NLR integrates two key components of the immune response: neutrophilia, which reflects systemic inflammatory activation, and lymphopenia, which indicates impaired adaptive immunity. Previous studies have consistently demonstrated the prognostic significance of NLR in a variety of solid tumors and hematologic malignancies. In patients with MM, elevated baseline NLR has been associated with adverse clinicopathological characteristics, inferior treatment response, and shorter progression-free and overall survival. However, the existing literature has focused predominantly on survival-related outcomes, whereas the potential relationship between baseline NLR and subsequent serious infectious complications has received considerably less attention. Therefore, the clinical utility of NLR in infection-risk assessment in MM remains incompletely defined [10,11,12,13,14].

In view of these considerations, improving the identification of patients at increased risk for serious infections represents an important unmet need in the contemporary management of multiple myeloma. Early recognition of high-risk individuals may facilitate timely implementation of preventive strategies and optimize supportive care. Therefore, the present study aimed to evaluate clinical and laboratory factors associated with serious infections in patients with multiple myeloma, with particular emphasis on the relationship between baseline inflammatory markers, including the neutrophil-to-lymphocyte ratio, and subsequent serious infectious complications in routine clinical practice.

## 2. Materials and Methods

### 2.1. Study Design and Patient Population

This multicenter retrospective cohort study included consecutive adult patients with newly diagnosed multiple myeloma (MM) who were treated and followed at participating hematology centers between January 2020 and December 2025. The diagnosis of MM was established according to the International Myeloma Working Group (IMWG) diagnostic criteria.

Patients aged ≥ 18 years with newly diagnosed MM and available baseline clinical and laboratory data at diagnosis were eligible for inclusion. Patients with active infection at diagnosis, incomplete medical records, or missing baseline laboratory data required for the study analyses were excluded.

### 2.2. Data Collection

Demographic, disease-related, treatment-related, laboratory, and follow-up data were retrospectively retrieved from electronic medical records.

The following baseline variables were recorded:

- Age at diagnosis;
- Sex;
- International Staging System (ISS) stage;
- Myeloma subtype;
- Bone disease;
- Dialysis requirement at diagnosis;
- Cytogenetic risk category (when available);
- First-line treatment regimen;
- Treatment intensity (triplet or quadruplet therapy);
- Autologous stem cell transplantation (ASCT);
- Maintenance therapy and maintenance regimen;
- Treatment response;
- Maintenance therapy after ASCT.

Baseline laboratory parameters obtained at diagnosis before initiation of anti-myeloma therapy included:

- C-reactive protein (CRP);
- Serum albumin;
- Serum creatinine;
- Estimated glomerular filtration rate (eGFR);
- Hemoglobin;
- Platelet count;
- Serum calcium;
- β_2_-microglobulin.

The neutrophil-to-lymphocyte ratio (NLR) was calculated from the absolute neutrophil and lymphocyte counts obtained from the same complete blood count sample at diagnosis.

Clinical follow-up data included IVIG administration, infectious complications, infection type, hypogammaglobulinemia, neutropenia, disease progression, survival status, progression-free survival (PFS), follow-up duration, and time to IVIG administration.

### 2.3. Definitions

The primary study outcome was the development of serious infection during follow-up. Serious infection was defined as any infectious episode requiring hospitalization, including but not limited to sepsis and pneumonia. Infectious events were recorded throughout the entire follow-up period, including those occurring during second-line and subsequent therapies in patients with relapsed or refractory disease. Dialysis requirement at diagnosis was recorded as a binary variable (yes/no). The decision to initiate dialysis was made by the treating nephrology team according to clinical assessment; however, the specific indications for dialysis initiation were not systematically available in the retrospective dataset.

Hypogammaglobulinemia was defined as a serum IgG level < 5 g/L during follow-up. In patients who developed serious infection, hypogammaglobulinemia considered in the analysis was documented before the first serious infectious event. Neutropenia was defined as an absolute neutrophil count < 1.5 × 10^9^/L during follow-up. IVIG administration was performed according to institutional clinical practice based on recurrent or frequent infections, hypogammaglobulinemia, and the treating physician’s clinical judgment. No uniform predefined criteria for IVIG initiation were applied across the study population.

Antimicrobial prophylaxis was administered according to institutional clinical practice and generally consisted of levofloxacin for antibacterial prophylaxis and acyclovir for antiviral prophylaxis. Routine antifungal prophylaxis was not administered.

Cytogenetic risk was classified according to available fluorescence in situ hybridization (FISH) findings. High-risk cytogenetics was defined by the presence of del(17p), t(4;14), t(14;16), and/or gain or amplification of 1q, whereas patients with available cytogenetic data but without these abnormalities were classified as standard risk. Patients for whom cytogenetic data could not be retrieved retrospectively were considered to have unavailable cytogenetic risk and were not included in risk-category comparisons.

Treatment response was assessed according to International Myeloma Working Group response criteria. Individual response categories were recorded, ranging from complete response (CR) to progressive disease (PD). For the present analysis, treatment response was dichotomized as achieved versus not achieved; achievement of at least a partial response (PR), including PR, very good partial response (VGPR), and CR, was considered a treatment response, whereas stable disease (SD) and progressive disease (PD) were categorized as no treatment response. For patients who developed a serious infection, the treatment response used in the analysis was the response status documented before the first serious infectious event.

### 2.4. Study Outcomes

The primary objective of this study was to evaluate clinical and laboratory factors associated with the development of serious infection in patients with multiple myeloma. Secondary analyses evaluated the relationship between baseline inflammatory markers, particularly the neutrophil-to-lymphocyte ratio, and serious infectious complications, as well as the associations of serious infection with supportive-care and clinical outcomes.

### 2.5. Statistical Analysis

Statistical analyses were performed using IBM SPSS Statistics version 25.0 (IBM Corp., Armonk, NY, USA).

Continuous variables were expressed as mean ± standard deviation (SD) or median (interquartile range [IQR]), according to data distribution, whereas categorical variables were presented as frequencies and percentages. The normality of continuous variables was assessed using the Kolmogorov–Smirnov test.

Comparisons between patients with and without serious infection were performed using Student’s *t*-test for normally distributed continuous variables and the Mann–Whitney U test for non-normally distributed variables. The distribution of categorical variables was compared using Pearson’s chi-square test or an exact chi-square test, as appropriate.

Associations with serious infection were initially evaluated using univariate binary logistic regression analysis, and the results were expressed as odds ratios (ORs) with corresponding 95% confidence intervals (CIs). A multivariable binary logistic regression model was subsequently constructed to evaluate factors independently associated with serious infection. Age, International Staging System (ISS) stage, dialysis requirement, treatment response, and baseline neutrophil-to-lymphocyte ratio (NLR) were included in the multivariable model based on their clinical relevance to infection risk and the objectives of the study. ISS stage was entered as a categorical variable, with stage I used as the reference category. Treatment response was dichotomized as achieved versus not achieved, as defined above.

Follow-up variables that could occur after or in response to infectious complications, including IVIG administration, hypogammaglobulinemia, disease progression, and mortality, were not included in the primary multivariable model to minimize potential temporal ambiguity and reverse causality. Infection type was also not included in the multivariable model because it represents the infectious event itself and complete separation occurred for sepsis, with all patients with sepsis belonging to the serious infection group.

All statistical tests were two-sided, and a *p*-value < 0.05 was considered statistically significant.

### 2.6. Ethical Approval

The study was conducted in accordance with the ethical principles of the Declaration of Helsinki. Ethical approval was obtained from the Ordu University Clinical Research Ethics Committee (Approval No. 2026/97; 10 April 2026). Owing to the retrospective design of the study, the requirement for written informed consent was waived by the Ethics Committee.

The study was reported in accordance with the Strengthening the Reporting of Observational Studies in Epidemiology (STROBE) statement.

## 3. Results

### 3.1. Patient Characteristics

A total of 104 patients with multiple myeloma were included in this multicenter retrospective study. The mean age at diagnosis was 64.5 ± 9.8 years (range, 37–85 years), and 57 (54.8%) patients were male. Bone disease was present in 88 (84.6%) patients, while at diagnosis, 15 patients (14.4%) required dialysis. According to the International Staging System (ISS), 32 (30.8%) patients had stage I disease, 36 (34.6%) had stage II disease, and 36 (34.6%) had stage III disease. IgG was the predominant myeloma subtype (63.5%), followed by IgA (18.3%), kappa light-chain (10.6%), and lambda light-chain (7.7%) disease (Table 1).

Regarding first-line therapy, the majority of patients received VCD (78.8%), whereas 18.3% received VRD, 1.9% received DRD, and 1.0% received DVD. Autologous stem cell transplantation (ASCT) was performed in 60 (57.7%) patients, and maintenance therapy was administered to 56 (53.8%), most commonly with lenalidomide (89.3%). Overall, treatment response was achieved in 79 (76.0%) patients.

During follow-up, 60 (57.7%) patients received intravenous immunoglobulin (IVIG), 80 (76.9%) experienced at least one infectious episode, and 39 (37.5%) developed serious infections requiring hospitalization. Hypogammaglobulinemia was observed in 38 (36.5%) patients, neutropenia in 49 (47.1%), and disease progression in 55 (52.9%). Overall mortality during follow-up was 26.9%. The mean follow-up duration was 52.1 ± 26.1 months, and the mean progression-free survival was 30.1 ± 15.9 months (Table 1).

### 3.2. Baseline Laboratory Findings

The baseline laboratory characteristics are summarized in Table 2. The mean serum C-reactive protein level was 12.45 ± 20.40 mg/L, while the mean albumin concentration was 3.82 ± 0.59 g/dL. The mean serum creatinine level was 1.10 ± 0.98 mg/dL, corresponding to a mean estimated glomerular filtration rate (eGFR) of 80.69 ± 20.94 mL/min/1.73 m^2^. The mean hemoglobin concentration was 10.53 ± 2.02 g/dL, and the mean platelet count was 222.3 ± 95.9 ×10^9^/L. The mean β_2_-microglobulin level was 5.46 ± 7.39 mg/L, the mean calcium concentration was 9.27 ± 1.14 mg/dL, and the mean baseline NLR was 2.74 ± 2.35, with a median of 2.17 (range, 0.18–16.00) (Table 2).

### 3.3. Comparison of Patients with and Without Serious Infection

The demographic, clinical, and treatment characteristics of patients according to the development of serious infection are presented in Table 3. Patients who developed serious infection had a significantly higher frequency of dialysis requirement than those without serious infection (30.8% vs. 4.6%, *p* = 0.001). Similarly, failure to achieve treatment response was markedly more common among patients with serious infection (51.3% vs. 7.7%, *p* < 0.001). IVIG administration was also significantly more frequent in the serious infection group (76.9% vs. 46.2%, *p* = 0.002).

Hypogammaglobulinemia was observed more frequently in patients with serious infection than in those without (48.7% vs. 29.2%, *p* = 0.046). Likewise, disease progression occurred significantly more often in patients who developed serious infection (69.2% vs. 43.1%, *p* = 0.010), and mortality was substantially higher in this group (59.0% vs. 7.7%, *p* < 0.001).

In an additional analysis according to IVIG use, disease progression occurred in 36 of 60 patients (60.0%) who received IVIG and in 19 of 44 patients (43.2%) who did not receive IVIG (*p* = 0.09). Mortality occurred in 17 of 60 patients (28.3%) who received IVIG and in 11 of 44 patients (25.0%) who did not receive IVIG (*p* = 0.71). Thus, neither progression nor mortality differed significantly according to IVIG use.

No significant differences were observed between patients with and without serious infection regarding age, sex, bone disease, ISS stage, myeloma subtype, first-line treatment regimen, autologous stem cell transplantation, cytogenetic risk, treatment intensity, maintenance therapy, maintenance regimen, maintenance after ASCT, duration of IVIG administration, antimicrobial prophylaxis, or neutropenia (all *p* > 0.05). Baseline eGFR was lower in patients with serious infection than in those without serious infection (73.86 ± 22.51 vs. 84.80 ± 18.79 mL/min/1.73 m^2^), although the difference did not reach statistical significance (*p* = 0.08).

Among the 39 patients who developed a serious infection requiring hospitalization, the recorded infection types were upper respiratory tract infection in 14 (35.9%), sepsis in 11 (28.2%), pneumonia in 9 (23.1%), catheter-related infection in 3 (7.7%), COVID-19 in 1 (2.6%), and febrile neutropenia in 1 (2.6%). Analysis of infection type demonstrated a significant difference between patients with and without serious infection (*p* < 0.001). Sepsis occurred exclusively in patients with serious infection (28.2% vs. 0%, *p* < 0.001), whereas upper respiratory tract infections were significantly more common among patients without serious infection (82.9% vs. 35.9%, *p* < 0.001). The frequencies of pneumonia, catheter-related infections, urinary tract infections, COVID-19, conjunctivitis, and febrile neutropenia did not differ significantly between the groups.

### 3.4. Comparison of Baseline Laboratory Parameters According to Serious Infection

Baseline laboratory findings according to the development of serious infection are summarized in Table 4. Patients with serious infection tended to have lower estimated glomerular filtration rates than those without serious infection (73.86 ± 22.51 vs. 84.80 ± 18.79 mL/min/1.73 m^2^); however, this difference did not reach statistical significance (*p* = 0.080).

No statistically significant differences were observed between the two groups with respect to serum albumin, C-reactive protein, creatinine, platelet count, β_2_-microglobulin, calcium, hemoglobin, or neutrophil-to-lymphocyte ratio (all *p* > 0.05).

### 3.5. Univariate Logistic Regression Analysis

**Figure 1:**
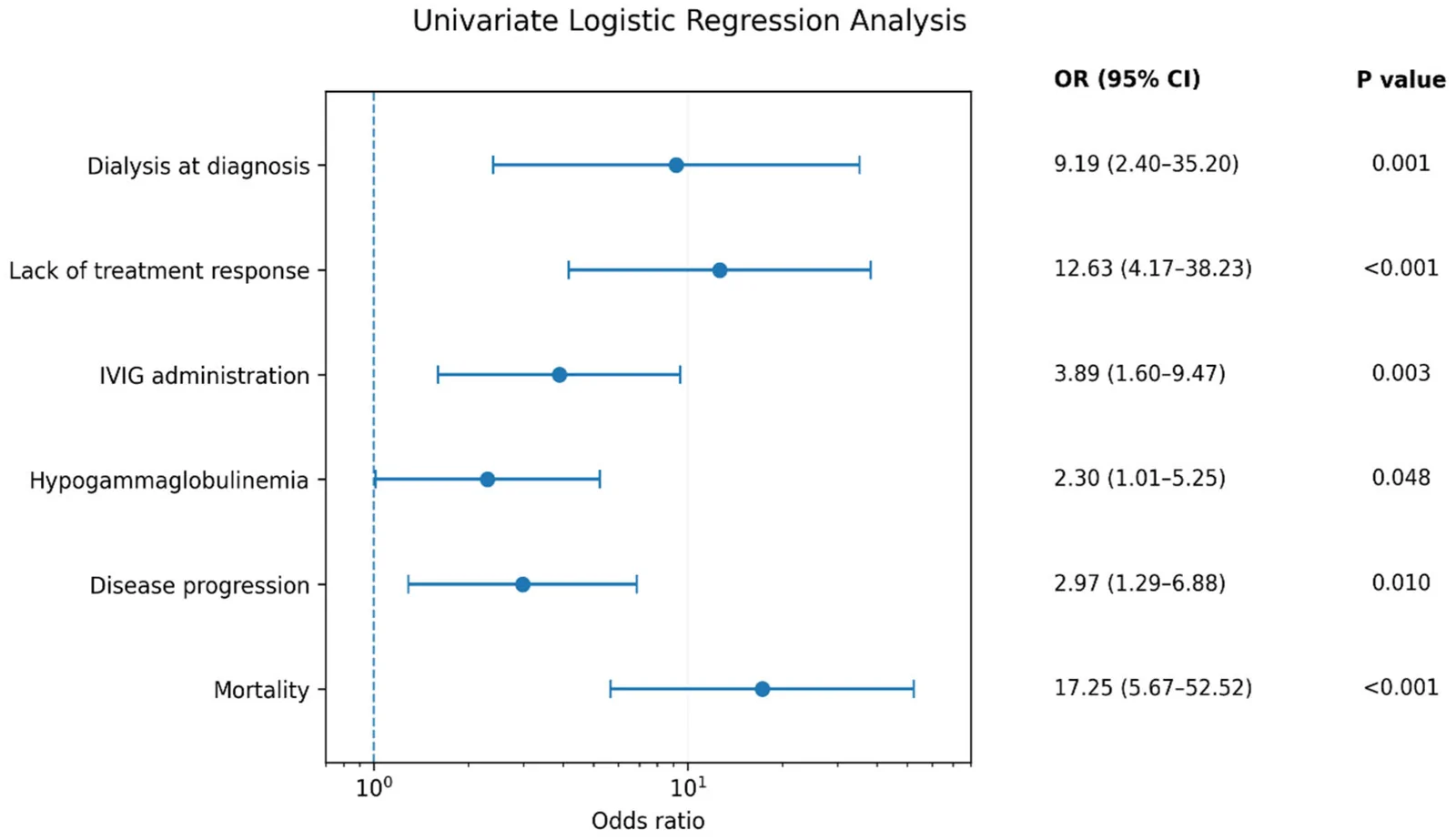
Forest plot of the univariate logistic regression analysis of factors associated with serious infection in patients with multiple myeloma. The vertical dashed line represents an odds ratio of 1. Error bars indicate 95% confidence intervals. Dialysis refers to dialysis requirement at diagnosis.

In univariate logistic regression analysis, dialysis requirement at diagnosis was associated with higher odds of serious infection (OR 9.19, 95% CI 2.40–35.20, *p* = 0.001). Failure to achieve treatment response showed a strong association with serious infection (OR 12.63, 95% CI 4.17–38.23, *p* < 0.001). IVIG administration (OR 3.89, 95% CI 1.60–9.47, *p* = 0.003), hypogammaglobulinemia (OR 2.30, 95% CI 1.01–5.25, *p* = 0.048), disease progression (OR 2.97, 95% CI 1.29–6.88, *p* = 0.010), and mortality (OR 17.25, 95% CI 5.67–52.52, *p* < 0.001) were also associated with serious infection in univariate analyses (Table 5 and Figure 1). These findings represent unadjusted associations and should not be interpreted as evidence of causality.

### 3.6. Multivariable Logistic Regression Analysis

**Figure 2:**
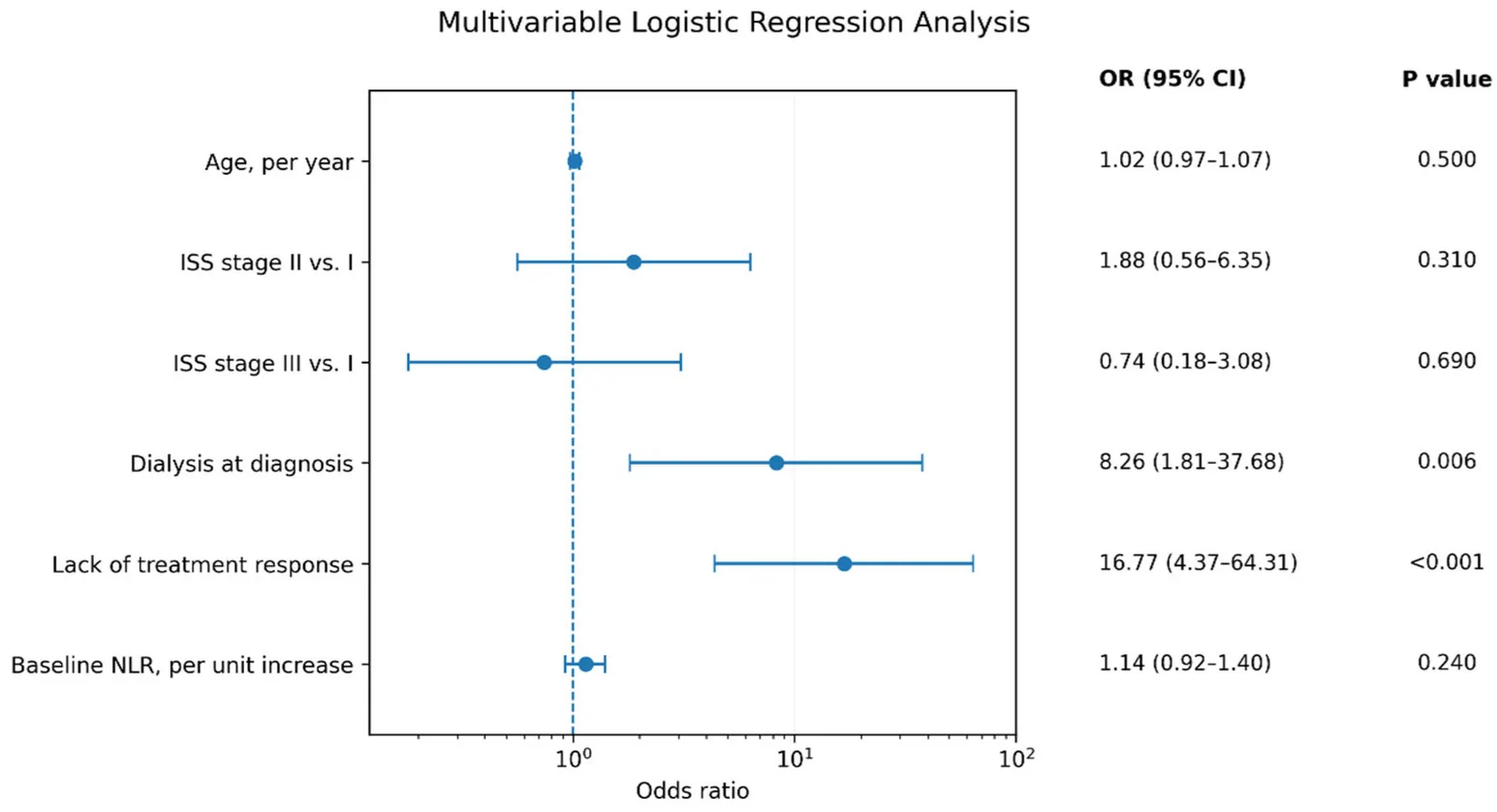
Forest plot of the multivariable logistic regression analysis of factors associated with serious infection. The vertical dashed line represents an odds ratio of 1. Error bars indicate 95% confidence intervals. ISS stage I was used as the reference category. Treatment response was considered achieved when at least a partial response (PR) was attained. Dialysis refers to dialysis requirement at diagnosis. The multivariable model included age, ISS stage, dialysis requirement at diagnosis, treatment response, and baseline neutrophil-to-lymphocyte ratio (NLR).

Factors independently associated with serious infection in the multivariable logistic regression analysis are presented in Table 6 and illustrated in Figure 2. After adjustment for age, ISS stage, dialysis requirement, treatment response, and baseline NLR, dialysis requirement at diagnosis remained independently associated with serious infection (adjusted OR 8.26, 95% CI 1.81–37.68, *p* = 0.006). Lack of treatment response showed an even stronger independent association with serious infection (adjusted OR 16.77, 95% CI 4.37–64.31, *p* < 0.001).

In contrast, age (adjusted OR 1.02, 95% CI 0.97–1.07, *p* = 0.500) and baseline NLR (adjusted OR 1.14, 95% CI 0.92–1.40, *p* = 0.240) were not independently associated with serious infection. Using ISS stage I as the reference category, neither ISS stage II (adjusted OR 1.88, 95% CI 0.56–6.35, *p* = 0.310) nor ISS stage III (adjusted OR 0.74, 95% CI 0.18–3.08, *p* = 0.690) showed a statistically significant independent association with serious infection.

Given the relatively small number of serious infection events (n = 39), a sensitivity analysis was performed using Firth penalized likelihood logistic regression. The results of this sensitivity analysis are presented in Table 7 and were consistent with the primary multivariable analysis. Dialysis requirement at diagnosis remained independently associated with serious infection (adjusted OR 6.70, 95% CI 1.76–31.76, *p* = 0.005), as did lack of treatment response (adjusted OR 12.91, 95% CI 4.02–50.71, *p* < 0.001), whereas baseline NLR remained not significantly associated with serious infection (adjusted OR 1.12, 95% CI 0.92–1.38, *p* = 0.239).

## 4. Discussion

This retrospective multicenter cohort study evaluated clinical and laboratory factors associated with serious infections requiring hospitalization in patients with multiple myeloma. Rather than identifying entirely novel risk factors, our findings provide real-world evidence that dialysis requirement at diagnosis and inadequate treatment response were independently associated with serious infection. The study also highlights the complex relationship between serious infection, IVIG use, disease progression, and mortality in routine clinical practice, while baseline NLR did not show an independent association with serious infection.

Despite substantial therapeutic advances achieved with proteasome inhibitors, immunomodulatory drugs, monoclonal antibodies, and other novel agents, infectious complications remain a major cause of hospitalization, treatment interruption, and mortality in patients with MM. Contemporary cohort studies have consistently demonstrated that clinically significant infections continue to occur frequently, particularly during the early phase of treatment, and remain an important determinant of adverse clinical outcomes despite improved disease control [15,16,17,18]. In our cohort, serious infections requiring hospitalization occurred in 37.5% of patients, further emphasizing that infection continues to represent a considerable clinical burden in routine practice. These findings reinforce the importance of early identification of patients at increased infectious risk and support the implementation of individualized supportive-care strategies. In this context, the TEAMM randomized trial demonstrated that antibacterial prophylaxis with levofloxacin significantly reduced early infectious complications in newly diagnosed MM, highlighting the clinical benefit of risk-adapted preventive approaches in appropriately selected high-risk patients [19].

Corticosteroid exposure represents an important treatment-related contributor to infectious risk in patients with MM. The immunosuppressive effects of corticosteroids are influenced by the intensity and duration of exposure, and higher or prolonged corticosteroid exposure may further increase susceptibility to infectious complications. In our cohort, most patients received weekly dexamethasone as part of their standard anti-myeloma treatment regimens, with doses generally ranging from 20 to 40 mg per week according to individual patient characteristics and comorbidities, including diabetes mellitus. Therefore, minimizing unnecessary corticosteroid exposure and tailoring steroid intensity to patient characteristics are important considerations in infection-risk mitigation [5]. However, individual cumulative corticosteroid exposure was not systematically recorded, precluding a direct comparison of infectious risk between lower- and higher-dose steroid exposure in the present study.

Another important finding of the present study was the significant association between IVIG administration and serious infection. At first glance, this observation may appear paradoxical; however, it should not be interpreted as indicating that IVIG increases the risk of infection. Rather, IVIG administration most likely reflects the selection of patients with advanced immune dysfunction, recurrent infections, or persistent hypogammaglobulinemia who inherently have a substantially higher baseline risk of infectious complications. Therefore, the observed association is more likely to represent the clinical characteristics of patients selected for immunoglobulin replacement than a detrimental effect of IVIG itself.

Current evidence supports the use of IVIG as an individualized supportive-care strategy rather than as routine prophylaxis for all patients with MM. Early evidence from the randomized study by Chapel et al. demonstrated that IVIG significantly reduced serious bacterial infections in selected patients with stable MM and recurrent infections, establishing the rationale for immunoglobulin replacement in carefully selected individuals [7]. More recently, contemporary real-world studies have confirmed that IVIG has been associated with a reduction in recurrent infections in patients with secondary hypogammaglobulinemia, particularly among those with repeated infectious episodes despite appropriate antimicrobial prophylaxis and disease-directed therapy [20]. In addition, current expert recommendations emphasize that IVIG administration should be individualized according to infection history, immunoglobulin levels, and overall immune status rather than based solely on laboratory-defined hypogammaglobulinemia [5,8,9]. Consistent with this approach, the higher frequency of IVIG administration among patients with serious infection in our cohort should be interpreted in the context of confounding by indication and potential reverse causality. Accordingly, IVIG administration was not included in the primary multivariable model evaluating factors independently associated with serious infection.

The increasing use of modern immunotherapies has further highlighted the importance of treatment-specific infection risk and immune monitoring in MM. Anti-CD38 monoclonal antibodies, particularly daratumumab-containing regimens, have been associated with progressive depletion of polyclonal immunoglobulins and increased susceptibility to respiratory tract infections [21]. Likewise, BCMA-directed bispecific antibodies are associated with particularly high rates of infectious complications in the setting of profound and sustained humoral immune dysfunction, and IVIG replacement has been associated with a marked reduction in serious infections in these highly immunocompromised patients [22]. These treatment-related risks emphasize the importance of individualized infection-mitigation strategies, including vaccination, appropriate antimicrobial prophylaxis, monitoring of immunoglobulin levels, early recognition and treatment of infections, and consideration of IVIG replacement in selected patients with recurrent infections and clinically significant hypogammaglobulinemia [5,8,9]. In our cohort, initial therapy was predominantly VCD-based. Although some patients may have received newer therapeutic approaches during subsequent lines of therapy, detailed data regarding subsequent treatment exposure were not systematically available for analysis. Therefore, the infection risks associated with contemporary treatment strategies, including quadruplet regimens, BCMA-directed bispecific antibodies, and CAR-T-cell therapy, could not be specifically evaluated, and the generalizability of our findings to patients receiving these approaches may be limited.

Hypogammaglobulinemia was significantly associated with serious infection in the univariate analysis. In patients who developed serious infection, hypogammaglobulinemia considered in the analysis was documented before the first serious infectious event. However, quantitative baseline and follow-up IgG levels were not systematically available, and hypogammaglobulinemia was recorded as a categorical variable based on a serum IgG level < 5 g/L. Therefore, changes in IgG concentrations over time and prognosis according to quantitative serum IgG levels could not be evaluated. Moreover, serum immunoglobulin levels alone may not adequately capture the complexity of immune dysfunction in MM, in which infectious vulnerability may reflect the combined effects of disease burden, treatment response, renal impairment, cumulative immunosuppression, and other clinical factors. These findings support a comprehensive clinical assessment when considering IVIG therapy rather than reliance on hypogammaglobulinemia alone.

Baseline NLR was not independently associated with serious infection in the present study. Although NLR is an inexpensive and readily available marker reflecting systemic inflammation and host immune status, its clinical utility in multiple myeloma has been investigated predominantly in relation to disease prognosis rather than infectious complications. Previous studies have associated elevated baseline NLR with advanced disease characteristics, inferior treatment response, and shorter progression-free and overall survival in patients with newly diagnosed MM [12,13,14]. However, evidence specifically addressing baseline NLR as a predictor of subsequent serious infections remains limited.

The lack of association observed in our cohort may reflect the dynamic nature of infection risk in MM. NLR was assessed only once at diagnosis and may not capture subsequent changes related to treatment, disease progression, immune reconstitution, or supportive care. In addition, serious infections arise from multiple interacting factors, including disease burden, humoral immune dysfunction, renal impairment, treatment-related immunosuppression, and host characteristics, which may not be adequately represented by a single baseline inflammatory marker. The relatively modest sample size may also have limited the ability to detect weaker associations. Accordingly, baseline NLR should not be interpreted as an isolated predictor of serious infection, and its potential role may require evaluation alongside broader clinical and immune parameters in larger prospective studies.

Among all evaluated variables, dialysis requirement at diagnosis emerged as one of the factors independently associated with serious infection. After adjustment for age, ISS stage, treatment response, and baseline NLR, patients requiring dialysis at diagnosis had approximately eightfold higher odds of serious infection than those not requiring dialysis (adjusted OR 8.26, 95% CI 1.81–37.68, *p* = 0.006). This finding is clinically plausible, as renal impairment in MM reflects not only advanced disease burden but also profound alterations in innate and adaptive immune function. Patients requiring dialysis are exposed to repeated vascular access, frequent healthcare contact, impaired neutrophil and complement function, and metabolic disturbances related to uremia, all of which may contribute to increased susceptibility to severe bacterial infections. Similar observations have been reported in previous cohort studies, in which renal dysfunction was associated with an increased risk of infectious complications and inferior clinical outcomes in patients with MM [16]. In our cohort, baseline eGFR was also lower among patients who subsequently developed serious infections than among those without serious infections (73.86 ± 22.51 vs. 84.80 ± 18.79 mL/min/1.73 m^2^), although this difference did not reach statistical significance (*p* = 0.080). This finding suggests that the observed association was most pronounced among patients with advanced renal dysfunction requiring dialysis rather than across the full spectrum of baseline renal impairment. In line with these findings, the International Myeloma Working Group recognizes renal impairment as a major determinant of clinical vulnerability and recommends prompt disease control together with individualized supportive care in this particularly high-risk population [23].

Failure to achieve treatment response showed the strongest independent association with serious infection in the multivariable model. After adjustment for age, ISS stage, dialysis requirement, and baseline NLR, patients who failed to achieve treatment response had approximately 17-fold higher odds of serious infection than responders (adjusted OR 16.77, 95% CI 4.37–64.31, *p* < 0.001). Persistent disease activity is accompanied by ongoing immunoparesis, suppression of normal immunoglobulin production, progressive bone marrow dysfunction, and prolonged exposure to anti-myeloma therapy, all of which may contribute to impaired host defense mechanisms. Therefore, the association between inadequate treatment response and serious infection is biologically plausible and emphasizes the importance of achieving rapid and durable disease control. Current therapeutic strategies increasingly aim to achieve deep hematologic responses not only to improve progression-free and overall survival but also to reduce disease-related complications and preserve immune function [1].

Collectively, these findings indicate that readily available clinical parameters reflecting renal dysfunction and treatment effectiveness may be useful for identifying patients at increased risk of serious infection, whereas a single baseline inflammatory biomarker such as NLR did not provide independent risk discrimination in this cohort. Consequently, patients requiring dialysis or failing to achieve an adequate treatment response may warrant closer clinical surveillance, early infection-prevention strategies, and individualized supportive-care interventions throughout the disease course.

The present study has several strengths. To our knowledge, this is one of the few real-world studies specifically evaluating both serious infections and IVIG administration in patients with multiple myeloma. The inclusion of demographic, disease-related, treatment-related, and laboratory variables enabled a comprehensive assessment of factors associated with serious infectious complications, while multivariable logistic regression allowed assessment of factors independently associated with serious infection after adjustment for potential confounding factors. In addition, the consistency of the main findings in the Firth penalized likelihood sensitivity analysis supports the robustness of the primary multivariable results despite the relatively modest number of serious infection events.

Several limitations should also be acknowledged. First, the retrospective multicenter design may have introduced selection and information bias and precludes the establishment of causal relationships. Second, the relatively modest sample size may have limited the statistical power to detect weaker associations, particularly for inflammatory biomarkers such as NLR. Third, baseline NLR was assessed only at diagnosis and did not account for dynamic changes in inflammatory or immune status during treatment. Although treatment response was documented before the first serious infectious event in patients who developed serious infection, the exact time interval from diagnosis or treatment initiation to the first serious infection was not collected as a separate analytical variable, limiting a more detailed assessment of the temporal relationship between treatment response and infection risk. In addition, IVIG administration was based on physician judgment and institutional clinical practice rather than predefined standardized criteria. Accordingly, the observed association between IVIG administration and serious infection most likely reflects confounding by indication rather than a causal relationship. This limitation is consistent with current recommendations advocating individualized patient selection for immunoglobulin replacement rather than universal administration [5,9,20,22]. The treatment landscape represented in our cohort primarily reflects initial therapy, which was predominantly VCD-based. Although some patients may have received newer therapeutic approaches during subsequent lines of therapy, these data were not systematically available for analysis. Therefore, the generalizability of our findings to patients receiving contemporary strategies, including quadruplet regimens, BCMA-directed bispecific antibodies, and CAR-T-cell therapy, may be limited. Although NLR was calculated from baseline complete blood count data, absolute neutrophil and lymphocyte counts were not retained as separate analytical variables. In addition, CD4+ T-cell counts and ferritin levels were not systematically available and therefore could not be evaluated as potential infection-related biomarkers. Recurrent serious infection events and ICU admissions were not systematically available in the retrospective dataset and therefore could not be reliably analyzed. Although first-line treatment regimens were available, the specific treatment regimen and line of therapy at the time of each serious infectious event were not systematically captured. Renal recovery following dialysis was not systematically recorded and therefore could not be evaluated. The specific cause of death could not be reliably determined from the retrospective medical records; therefore, infection-attributable mortality could not be separately assessed. Quantitative serum IgG levels were not available in the dataset; hypogammaglobulinemia was recorded only as a categorical variable, which precluded a detailed analysis of prognosis according to serum IgG levels.

Despite these limitations, our findings have important clinical implications. Early identification of patients at increased risk of serious infection may facilitate individualized supportive-care strategies, optimize the use of antimicrobial prophylaxis and immunoglobulin replacement, and improve clinical outcomes. These observations are consistent with the growing emphasis on risk-adapted infection prevention strategies in contemporary MM management [24].

Taken together, our findings emphasize that clinical assessment incorporating renal function, treatment response, and immune status may provide greater value for infection risk stratification than reliance on a single baseline inflammatory biomarker. Further prospective studies incorporating longitudinal immune and treatment-related variables are warranted to better define individualized strategies for infection-risk assessment and prevention in patients with multiple myeloma.

## 5. Conclusions

Serious infections remain a frequent and clinically important complication in patients with multiple myeloma despite major advances in anti-myeloma therapy. Dialysis requirement at diagnosis and failure to achieve treatment response were independently associated with serious infection, whereas baseline NLR was not significantly associated with serious infectious complications in our cohort. These findings suggest that readily available clinical characteristics reflecting renal dysfunction and treatment effectiveness may be more informative than a single baseline inflammatory biomarker for identifying patients at increased risk of serious infection. Future prospective multicenter studies are warranted to validate these findings and to develop standardized risk-adapted models that facilitate early identification of patients at increased risk of serious infection and optimize supportive-care strategies, including appropriate patient selection for IVIG therapy.

## Figures and Tables

**Table 1:** Baseline demographic, clinical, and treatment characteristics (n = 104).

Characteristic		Overall (n = 104)
Age at diagnosis, years, mean ± SD (range)		64.5 ± 9.8 (37–85)
Sex, n (%)	Male	57 (54.8)
	Female	47 (45.2)
Bone disease, n (%)	No	16 (15.4)
	Yes	88 (84.6)
Dialysis at diagnosis, n (%)	No	89 (85.6)
	Yes	15 (14.4)
ISS stage, n (%)	I	32 (30.8)
	II	36 (34.6)
	III	36 (34.6)
Myeloma subtype, n (%)	Kappa light chain	11 (10.6)
	Lambda light chain	8 (7.7)
	IgG	66 (63.5)
	IgA	19 (18.3)
First-line treatment regimen, n (%)	VCD	82 (78.8)
	VRD	19 (18.3)
	DRD	2 (1.9)
	DVD	1 (1.0)
Autologous stem cell transplantation, n (%)	No	44 (42.3)
	Yes	60 (57.7)
Cytogenetic risk, n (%) *	Standard risk	49 (79.0)
	High risk	13 (21.0)
Treatment intensity, n (%)	Triplet therapy	103 (99.0)
	Quadruplet therapy	1 (1.0)
Maintenance therapy, n (%)	No	48 (46.2)
	Yes	56 (53.8)
Maintenance regimen, n (%)	Lenalidomide	50 (89.3)
	Bortezomib	2 (3.6)
	Combination	3 (5.4)
	Pomalidomide	1 (1.8)
Treatment response, n (%)	Achieved	79 (76.0)
	Not achieved	25 (24.0)
Maintenance after ASCT, n (%)	No	45 (43.3)
	Yes	59 (56.7)
IVIG administration, n (%)	No	44 (42.3)
	Yes	60 (57.7)
Any infection during follow-up, n (%)	No	24 (23.1)
	Yes	80 (76.9)
Type of infection, n (%)	Upper respiratory tract infection	48 (60.8)
	Sepsis	11 (14.0)
	Pneumonia	11 (13.9)
	Catheter-related infection	3 (3.8)
	Urinary tract infection	3 (3.8)
	COVID-19	1 (1.3)
	Conjunctivitis	1 (1.3)
	Febrile neutropenia	1 (1.3)
Serious infection requiring hospitalization, n (%)	No	65 (62.5)
	Yes	39 (37.5)
Hypogammaglobulinemia, n (%)	Yes	38 (36.5)
	No	66 (63.5)
Antimicrobial prophylaxis, n (%)	Yes	98 (94.2)
	No	6 (5.8)
Neutropenia, n (%)	Yes	49 (47.1)
	No	55 (52.9)
Disease progression, n (%)	Yes	55 (52.9)
	No	49 (47.1)
Vital status, n (%)	Alive	76 (73.1)
	Dead	28 (26.9)
Follow-up duration, months, mean ± SD (range)	52.1 ± 26.1 (3–156)	
Progression-free survival, months, mean ± SD (range)	30.1 ± 15.9 (3–126)	
Time to IVIG administration, months, mean ± SD (range)	13.9 ± 4.8 (4–60)	

Data are presented as mean ± standard deviation (SD) with minimum–maximum values in parentheses for continuous variables and number (percentage) for categorical variables. * Cytogenetic risk was available only for patients who underwent cytogenetic evaluation; percentages were calculated among patients with available cytogenetic data. Abbreviations: ISS, International Staging System; VCD, bortezomib-cyclophosphamide-dexamethasone; VRD, bortezomib-lenalidomide-dexamethasone; DRD, daratumumab-lenalidomide-dexamethasone; DVD, daratumumab-bortezomib-dexamethasone; ASCT, autologous stem cell transplantation; IVIG, intravenous immunoglobulin.

**Table 2:** Baseline laboratory characteristics at diagnosis (n = 104).

Variable	Overall (n = 104)
C-reactive protein, mg/L	12.45 ± 20.40 (0.05–148.00)
Albumin, g/dL	3.82 ± 0.59 (2.27–5.10)
Creatinine, mg/dL	1.10 ± 0.98 (0.40–7.47)
Platelet count, ×10^9^/L	222.3 ± 95.9 (57.0–694.0)
β_2_-microglobulin, mg/L	5.46 ± 7.39 (1.30–51.00)
Calcium, mg/dL	9.27 ± 1.14 (7.40–15.50)
Hemoglobin, g/dL	10.53 ± 2.02 (7.20–16.40)
Neutrophil-to-lymphocyte ratio	2.74 ± 2.35 (0.18–16.00)

Data are presented as mean ± standard deviation (SD), with minimum and maximum values in parentheses.

**Table 3:** Comparison of demographic, clinical, and treatment characteristics according to the development of serious infection (n = 104).

Characteristic	Category	Serious Infection Yes (n = 39)	No Serious Infection (n = 65)	*p* Value
Age at diagnosis, years, mean ± SD		65.6 ± 10.1	63.9 ± 9.7	0.38 *
Sex, n (%)	Male	19 (48.7)	38 (58.5)	0.42 †
	Female	20 (51.3)	27 (41.5)	
Bone disease, n (%)	Yes	35 (89.7)	53 (81.5)	0.26 †
	No	4 (10.3)	12 (18.5)	
Dialysis at diagnosis, n (%)	No	27 (69.2)	62 (95.4)	0.001 †
	Yes	12 (30.8)	3 (4.6)	
ISS stage, n (%)	I	11 (28.2)	21 (32.3)	0.14 †
	II	10 (25.6)	26 (40.0)	
	III	18 (46.2)	18 (27.7)	
Myeloma subtype, n (%)	Kappa light chain	5 (12.8)	6 (9.2)	0.26 †
	Lambda light chain	4 (10.3)	4 (6.2)	
	IgG	20 (51.3)	46 (70.8)	
	IgA	10 (25.6)	9 (13.8)	
First-line treatment regimen, n (%)	VCD	30 (76.9)	52 (80.0)	0.79 †
	VRD	7 (17.9)	12 (18.5)	
	DRD	1 (2.6)	1 (1.5)	
	DVD	1 (2.6)	0	
Autologous stem cell transplantation, n (%)	No	18 (46.2)	26 (40.0)	0.54 †
	Yes	21 (53.8)	39 (60.0)	
Cytogenetic risk, n (%)	Standard risk	16 (80.0)	33 (78.6)	0.99 †
	High risk	4 (20.0)	9 (21.4)	
Treatment intensity, n (%)	Triplet therapy	38 (97.4)	65 (100.0)	0.38 †
	Quadruplet therapy	1 (2.6)	0	
Maintenance therapy, n (%)	No	18 (46.2)	30 (46.2)	0.99 †
	Yes	21 (53.8)	35 (53.8)	
Maintenance regimen, n (%)	Lenalidomide	18 (85.7)	32 (91.4)	0.87 †
	Bortezomib	1 (4.8)	1 (2.9)	
	Combination	2 (9.5)	1 (2.9)	
	Pomalidomide	0	1 (2.9)	
Treatment response, n (%)	Not achieved	20 (51.3)	5 (7.7)	<0.001 †
	Achieved	19 (48.7)	60 (92.3)	
Maintenance after ASCT, n (%)	No	18 (46.2)	27 (41.5)	0.65 †
	Yes	21 (53.8)	38 (58.5)	
IVIG administration, n (%)	Yes	30 (76.9)	30 (46.2)	0.002 †
	No	9 (23.1)	35 (53.8)	
Time to IVIG administration, months, mean ± SD		14.3 ± 4.8	13.5 ± 5.5	0.73 *
Type of infection, n (%)				<0.001 †
	Upper respiratory tract infection	14 (35.9)	34 (82.9)	<0.001 ‡
	Sepsis	11 (28.2)	0	<0.001 ‡
	Pneumonia	9 (23.1)	3 (7.3)	0.06 ‡
	Catheter-related infection	3 (7.7)	0	0.07 ‡
	Urinary tract infection	0	3 (7.3)	0.09 ‡
	COVID-19	1 (2.6)	0	0.30 ‡
	Conjunctivitis	0	1 (2.4)	0.33 ‡
	Febrile neutropenia	1 (2.6)	0	0.30 ‡
Hypogammaglobulinemia, n (%)	Yes	19 (48.7)	19 (29.2)	0.046 †
	No	20 (51.3)	46 (70.8)	
Antimicrobial prophylaxis, n (%)	Yes	35 (89.7)	63 (96.9)	0.19 †
	No	4 (10.3)	2 (3.1)	
Neutropenia, n (%)	Yes	23 (59.0)	26 (40.0)	0.07 †
	No	16 (41.0)	39 (60.0)	
Disease progression, n (%)	Yes	27 (69.2)	28 (43.1)	0.010 †
	No	12 (30.8)	37 (56.9)	
Vital status, n (%)	Dead	23 (59.0)	5 (7.7)	<0.001 †
	Alive	16 (41.0)	60 (92.3)	

Data are presented as mean ± standard deviation (SD) or number (percentage), as appropriate. * Student’s *t*-test. † Pearson’s chi-square test or chi-square exact test, as appropriate. ‡ Exact comparison for individual infection categories. Abbreviations: ISS, International Staging System; VCD, bortezomib-cyclophosphamide-dexamethasone; VRD, bortezomib-lenalidomide-dexamethasone; DRD, daratumumab-lenalidomide-dexamethasone; DVD, daratumumab-bortezomib-dexamethasone; ASCT, autologous stem cell transplantation; IVIG, intravenous immunoglobulin.

**Table 4:** Comparison of baseline laboratory parameters between patients with and without serious infection (n = 104).

Variable	Serious Infection (n = 39)	No Serious Infection (n = 65)	*p* Value
Albumin, g/dL, mean ± SD	3.83 ± 0.62	3.81 ± 0.58	0.84 *
C-reactive protein, mg/L, median (IQR)	3.00 (1.50–12.00)	4.65 (3.00–13.75)	0.41 †
Creatinine, mg/dL, median (IQR)	0.90 (0.61–1.39)	0.86 (0.68–1.00)	0.29 †
Platelet count, ×10^9^/L, median (IQR)	206.0 (148.0–294.0)	227.0 (160.5–283.5)	0.42 †
β_2_-microglobulin, mg/L, median (IQR)	3.50 (2.33–6.05)	3.50 (2.60–4.45)	0.54 †
Calcium, mg/dL, mean ± SD	9.39 ± 1.29	9.20 ± 1.05	0.41 *
Hemoglobin, g/dL, mean ± SD	10.18 ± 1.77	10.75 ± 2.13	0.17 *
Neutrophil-to-lymphocyte ratio, mean ± SD	3.08 ± 2.64	2.54 ± 1.88	0.25 *
Estimated glomerular filtration rate, mL/min/1.73 m^2^, mean ± SD	73.86 ± 22.51	84.80 ± 18.79	0.08 *

Data are presented as mean ± standard deviation (SD) or median (interquartile range [IQR]), as appropriate. * Student’s *t*-test. † Mann–Whitney U test. Abbreviations: CRP, C-reactive protein; eGFR, estimated glomerular filtration rate; IQR, interquartile range; NLR, neutrophil-to-lymphocyte ratio.

**Table 5:** Univariate logistic regression analysis of factors associated with serious infection.

Variable	Odds Ratio (OR)	95% CI	*p* Value
Dialysis at diagnosis	9.19	2.40–35.20	0.001
Lack of treatment response	12.63	4.17–38.23	<0.001
IVIG administration	3.89	1.60–9.47	0.003
Infection type *	—	—	—
Hypogammaglobulinemia	2.30	1.01–5.25	0.048
Disease progression	2.97	1.29–6.88	0.010
Mortality	17.25	5.67–52.52	<0.001

Abbreviations: OR, odds ratio; CI, confidence interval; IVIG, intravenous immunoglobulin. Reported ORs represent unadjusted associations. * Logistic regression analysis for infection type could not be performed because of complete (perfect) separation. All patients who developed sepsis belonged to the serious infection group, resulting in failure of model estimation.

**Table 6:** Multivariable logistic regression analysis of factors associated with serious infection.

Variable	Adjusted OR	95% CI	*p* Value
Age, per year	1.02	0.97–1.07	0.500
ISS stage I	1.00 (reference)	—	—
ISS stage II vs. I	1.88	0.56–6.35	0.310
ISS stage III vs. I	0.74	0.18–3.08	0.690
Dialysis at diagnosis (yes vs. no)	8.26	1.81–37.68	0.006
Treatment response (no vs. yes)	16.77	4.37–64.31	<0.001
Baseline NLR, per unit increase	1.14	0.92–1.40	0.240

Abbreviations: OR, odds ratio; CI, confidence interval; ISS, International Staging System; NLR, neutrophil-to-lymphocyte ratio. ISS stage I was used as the reference category. Treatment response was considered achieved when at least a partial response (PR) was attained. Adjusted ORs were obtained from a multivariable binary logistic regression model including age, ISS stage, dialysis requirement at diagnosis, treatment response, and baseline NLR.

**Table 7:** Firth penalized likelihood logistic regression sensitivity analysis of factors associated with serious infection.

Variable	Adjusted OR	95% CI	*p* Value
Age, per year	1.02	0.97–1.07	0.52
ISS stage I	1.00 (reference)	—	—
ISS stage II vs. I	1.77	0.57–5.89	0.33
ISS stage III vs. I	0.78	0.19–2.91	0.714
Dialysis at diagnosis (yes vs. no)	6.70	1.76–31.76	0.005
Treatment response (no vs. yes)	12.91	4.02–50.71	<0.001
Baseline NLR, per unit increase	1.12	0.92–1.38	0.239

Abbreviations: OR, odds ratio; CI, confidence interval; ISS, International Staging System; NLR, neutrophil-to-lymphocyte ratio. The 95% CIs and *p*-values were obtained using the penalized profile-likelihood method.

## Data Availability

The datasets generated and/or analyzed during the current study are available from the corresponding author on reasonable request.
